# Opportunities and Challenges in HIV Treatment as Prevention Research: Results from the ANRS 12249 Cluster-Randomized Trial and Associated Population Cohort

**DOI:** 10.1007/s11904-020-00487-1

**Published:** 2020-02-18

**Authors:** Frank Tanser, Hae-Young Kim, Alain Vandormael, Collins Iwuji, Till Bärnighausen

**Affiliations:** 1 Lincoln Institute for Health, University of Lincoln, Lincoln, LN6 7TS UK; 2 Africa Health Research Institute, KwaZulu-Natal, South Africa; 3 School of Nursing and Public Health, University of KwaZulu-Natal, Durban, South Africa; 4 Centre for the AIDS Programme of Research in South Africa (CAPRISA), University of KwaZulu-Natal, Durban, South Africa; 5 Department of Population Health, New York University School of Medicine, New York, USA; 6 KwaZulu-Natal Innovation and Sequencing Platform, Durban, KwaZulu-Natal South Africa; 7 Heidelberg Institute of Global Health (HIGH), Faculty of Medicine and University Hospital, University of Heidelberg, Heidelberg, Germany; 8 Brighton and Sussex Medical School, University of Sussex, Brighton, UK; 9 Department of Global Health and Population, Harvard T.H. Chan School of Public Health, Boston, MA USA

**Keywords:** HIV, HIV prevention, HIV incidence, Antiretroviral therapy, Treatment as prevention, Population health, South Africa

## Abstract

**Purpose of Review:**

The ANRS 12249 treatment as prevention (TasP) trial investigated the impact of a universal test and treat (UTT) approach on reducing HIV incidence in one of the regions of the world most severely affected by the HIV epidemic—KwaZulu-Natal, South Africa. We summarize key findings from this trial as well as recent findings from controlled studies conducted in the linked population cohort quantifying the long-term effects of expanding ART on directly measured HIV incidence (2004–2017).

**Recent Findings:**

The ANRS TasP trial did not—and could not—demonstrate a reduction in HIV incidence, because the offer of UTT in the intervention communities did not increase ART coverage and population viral suppression compared to the standard of care in the control communities. Ten controlled studies from the linked population cohort—including several quasi-experimental study designs—exploit heterogeneity in ART exposure to show a consistent and substantial impact of expanding provision of ART and population viral suppression on reduction in HIV incidence at the couple, household, community, and population levels.

**Summary:**

In this setting, all of the evidence from large, population-based studies (inclusive of the ANRS TasP trial) is remarkably coherent and consistent—i.e., higher ART coverage and population viral suppression were repeatedly associated with clear, measurable decreases in HIV incidence. Thus, the expanded provision of ART has plausibly contributed in a major way toward the dramatic 43% decline in population-level HIV incidence in this typical rural African population. The outcome of the ANRS TasP trial constitutes a powerful null finding with important insights for overcoming implementation challenges in the population delivery of ART. This finding does not imply lack of ART effectiveness in blocking onward transmission of HIV nor its inability to reduce HIV incidence. Rather, it demonstrates that large increases in ART coverage over current levels will require health systems innovations to attract people living with HIV in early stages of the disease to participate in HIV treatment. Such innovations and new approaches are required for the true potential of UTT to be realized.

## Introduction

In 2018, approximately 38 million people worldwide were living with HIV [1]. About 80% of people living with HIV knew their status and nearly 80% of these people (23.3 million) accessed antiretroviral therapy (ART), a threefold increase from 2010. Despite the successful scale-up and access to HIV testing and treatment, HIV incidence remains high in many settings, with an estimated 1.7 million newly infected people in 2018. In particular, Eastern and Southern Africa remain the regions the most severely affected by the HIV epidemic [1].

The results from the landmark HPTN 052 trial in 2011 unequivocally showed that immediate initiation of ART was associated with a 96% reduction in HIV sexual transmission in sero-discordant stable couples [2]. To provide empirical evidence of the feasibility and effectiveness of a universal test and treat (UTT) strategy on reducing HIV incidence at the population level, four major community-based trials were initiated in Eastern and Southern Africa [3••, 4–6]. The first of these trials, the ANRS 12249 treatment as prevention (TasP) trial, was conducted in rural South Africa between 2012 and 2016 and offered home-based HIV testing and universal Art regardless of CD4 count in the intervention communities [3••, 7]. Three additional trials, BCPP (Botswana Combination Prevention Project) *-Ya Tsie*, PopART (Population Effects of Antiretroviral Therapy to Reduce HIV Transmission- HPTN071), and SEARCH (the Sustainable East Africa Research in Community Health) trials, were initiated in 2013 and completed between 2017 and 2018. The BCPP trial was a pair-matched community-randomized trial conducted in 30 communities and compared the standard of care in the control clusters with a combination prevention package in the intervention clusters (community mobilization, community-wide home-based and mobile HIV testing, targeted outreach testing men and women ≤ 25 years of age, active tracing and linkage to care support, increased access to male circumcision services, and expanded ART) [4]. PopART was conducted in 21 communities in Zambia and South Africa with three arms: Arm A: universal ART coupled with combination prevention intervention (door-to-door rapid HIV testing services, referral for voluntary medical male circumcision (VMMC) among uncircumcised HIV-negative men and antenatal care among HIV-positive pregnant women, screening and referral for tuberculosis (TB) and sexually transmitted infections (STIs), condom promotion and distribution) Arm B: ART provided according to local guidelines with combination prevention intervention and Arm C: the standard of care [5]. The SEARCH trial was a pair-matched cluster-randomized trial in 32 communities in rural Uganda and Kenya and included 2-week mobile, multi-disease, community health campaigns including rapid HIV testing, referral to HIV care, and home-based testing in all communities (i.e., both control and intervention arms) at baseline. Thereafter, the control communities received the standard of care (i.e., national guideline–restricted ART) while the intervention communities received annual repeat campaigns including HIV testing coupled with universal ART [6].

The outcomes and results of these trials have been well documented and described [8–13]. Briefly, two of the trials were able to demonstrate some evidence of moderate reduction in HIV incidence in intervention communities relative to the standard of care [4, 5]. In the BCPP, the HIV incidence was approximately 30% lower in the intervention communities (0.59 per 100 person-years vs. 0.92 per 100 person-years in the control communities) [4]. In the PopART, the HIV incidence in the arm which received combination prevention packages with ART administered according to national treatment guidelines (1.06 per 100 person-years) was 30% lower than that in the control arm (1.55 per 100 person-years), but there was no difference in the arm which received combination prevention packages in addition to universal ART (1.45 per 100 person-years) [5]. However, collectively, the four trials were unable to demonstrate consistent and substantial population reductions in HIV incidence. Aside from issues such as sexual mixing of populations which are clearly important [14], the more fundamental reason for lack of consistency in these findings is that many of the trials were unable induce a substantially higher ART coverage in intervention communities over the duration of the trial. Without a strong gradient in ART coverage across the trial arms, the causal effect of ART on population incidence cannot be estimated. Achieving such a coverage differential was made particularly difficult by the rapidly evolving treatment guidelines over the course of the trials (which resulted in control communities adopting the treat-all approach in three of the four trials over the course of participant follow-up) and in many cases due to exemplary care packages being delivered in “control communities”. Some of the trials (most notably SEARCH [6]) were highly successful in initiating large numbers of patients onto ART in both the intervention and control communities through an innovative community-based testing approach [6].

The ANRS TasP trial was conducted in the KwaZulu-Natal province of South Africa, a region considered by many to be at the epicentre of the HIV pandemic. The setting provides a remarkable opportunity to study the long-term impacts of ART scale-up on HIV incidence from within the same population because it also includes a linked population-based cohort which has been running for over 14 years. The population cohort is based on a very similar *modus operandi* to the ANRS TasP trial and uses the gold-standard approach of actively enrolling and following up a complete population observing individual HIV seroconversions in those who were initially observed to be HIV-uninfected. Here, we summarize key design features and results from the ANRS TasP trial as well as recent findings from ten controlled studies from the population-based cohort that directly quantified the long-term effects of expanding ART on directly measured HIV incidence. Several of the studies used strong quasi-experimental designs (such as regression discontinuity and instrumental variable designs), which, like randomized controlled trials, can control for both observed and unobserved confounding.

### Overview of the ANRS 12249 Cluster-Randomized Trial

The design of the ANRS TasP trial has been described in detail elsewhere [15, 16]. Briefly, the ANRS TasP trial evaluated the hypothesis that home-based HIV testing coupled with an immediate offer of ART would result in a decrease in population-level HIV incidence in a hyperendemic rural population. This hypothesis was tested in a two-arm cluster-randomized trial implemented between March 2012 and June 2016. Eleven control communities were offered ART according to standard of care (initially CD4 counts ≤ 350 cells/ml and then < 500 cells/ml from Jan 2015) and 11 intervention communities were offered ART regardless of CD4 count. The study was 80% powered to detect an overall 34% reduction in cumulative HIV incidence, with an estimated incidence of 2.25% per year in the control clusters over the trial period. The calculation explicitly considered the different lengths of follow-up time in the clusters, loss to follow-up, and the likelihood of re-testing of participants as well as the potential diluting effects of inter-cluster sexual mixing [15].

The ANRS TasP trial was the first of the four treatments as prevention trials and incorporated some novel features aimed at enhancing efficiency and delivery of the intervention in at least four areas are highlighting here. Firstly, other than expanded ART eligibility in the intervention arm, the interventions and mode of delivery were identical in both arms of the trial. In the later trials—BCPP, SEARCH, and PopART [4–6]—the makeup of the interventions differed from the control arms in ways other than just expanded ART eligibility, such that the trials evaluated the impact of a combination of interventions versus the standard of care rather than only the additional impact of UTT on population-level HIV incidence. In other words, these subsequent trials included additional or enhanced services in the intervention arm, besides universal ART. In the BCPP, these included enhanced community mobilization and expanded health prevention/screening, including male circumcision, distribution of condoms, and home-based HIV testing as well as HIV testing in mobile units during the community campaign [4]. In PopART study, specific mobile activities in the community, health screening for TB and STIs, and home-based HIV testing were offered in the intervention arms [5] while the SEARCH trial provided repeat annual community health campaigns or mobilization (including HIV testing at mobile sites, home-based HIV testing, and referral to HIV care) [6] for 3 years after the services were offered once in all intervention and control communities at baseline.

Secondly, the ANRS TasP trial (along with the SEARCH trial) evaluated the primary endpoint of HIV incidence among the whole trial population as opposed to a nested sub-sample of individuals within each cluster. Thirdly, the ANRS TasP trial used explicit linkage to records from the pre-existing public sector ART and trial clinics to quantify trends in ART coverage. This enabled robust calculation and comparison of the ART coverage at baseline and over the course of the trial in a way unaffected by the biases commonly associated with treatment self-report. Finally, ART was provided to participants in trial-specific clinics located in each of the 22 clusters at convenient locations. Many of the existing public sector clinics required long travelling and waiting times to receive treatment and care. Thus, trial clinics provided relatively easy access to treatment as each trial participant lived within 45 min’ walk of the clinic in their respective clusters.

### Results of the ANRS 12249 Cluster-Randomized Trial

During the trial period, 26,518 of 28,419 (93%) eligible individuals were contacted. Overall, there were 503 seroconversions documented after 22,891 person-years of follow-up. The trial team conducted testing and follow-up for an average of 2.3 years in each cluster. Over the course of the trial, the incidence in the control clusters was almost identical to the incidence that had been assumed in the sample size calculations, but did not differ significantly across the two arms: 2.11 per 100 person-years (95% CI 1.84–2.39) in the intervention arm versus 2.27 per 100 person-years in the control arm (95% CI 2.00–2.54) (adjusted hazard ratio 1.01, 95% CI 0.87–1.17). During the trial, more than 90% of HIV-positive individuals became aware of their diagnosis. However, at the end of the trial, there were no significant differences in both ART coverage and population viral suppression between the intervention and control communities. At the end of the trial, ART coverage was 52.8% in the control communities versus 53.4% in the intervention communities [3••]. Similarly, population levels of viral suppression were 46.2% versus 44.2% in the intervention versus control communities, from the baseline of 23.5% and 26.0%, respectively [17].

### Key Insights from the ANRS 12249 Cluster-Randomized Trial

The outcome of this well-conducted trial constitutes a powerful null finding with important lessons for overcoming challenges in the population delivery of ART. We highlight three key insights below. Firstly, the concern by participants about inadvertent disclosure of HIV status by attending one of the trial clinics likely contributed to the relatively poor linkage to care observed in the trial. Poor linkage to care was associated with being newly diagnosed with HIV, being students, living farther away from the clinics, or having higher educational attainment [18, 19]. The results brought into sharp focus the continued stigma around HIV and highlighted the critical need to normalize its treatment. A typical quote from a participant in this trial illustrates this point:

There are those who are still not keen [to attend the TasP clinic]. They have a problem that they will be seen at the park home [TasP clinic] and they say that the park home is full of people who have HIV. You see it is something like that. You see there are people who go to the clinic not because they are going to check their own illnesses but they keep looking at the people who are going to the research clinic and they say we are even carrying babies who have HIV. Now when a lot of people think about that they think if you go to that clinic you are visible, they wish they can hide from others. (Female, 51 years)

In this vein, the SEARCH trial model (described in detail elsewhere [20]) of taking a community-based, multi-disease approach for the management and treatment of HIV would seem to hold considerable promise.

Secondly, the contact rate was significantly lower in men and younger individuals [3••, 21]; however, among those who received the community intervention, linkage to care was similar in both men and women [18]. It is therefore vital that novel methods are found to engage men and younger populations to facilitate increased and more rapid linkage to treatment and care. In response to these findings, a 2 × 2 factorial cluster-randomized community-based trial, Home-Based Intervention to Test and Start (HITS) [22], was initiated in the AHRI population cohort [22, 23]. The HITS trial aims to establish the impact of small once-off financial incentives and a male-targeted HIV-specific decision support application on improving the uptake of HIV testing and linkage to care among men, with the ultimate aim of reducing population-level HIV incidence in (particularly young) women. Thus far, the HITS trial has demonstrated that a once-off financial micro-incentive of just $3 increased the uptake of HIV testing more than 50% among men [24].

Thirdly, the ANRS TasP trial identified individuals earlier in the course of their HIV infection, the majority of whom were asymptomatic. Competing priorities between livelihood sustenance, as seen by the high prevalence of food insecurity [25] in the trial population and time required to seek care, meant HIV-positive individuals likely delayed starting ART. Studies which have highlighted the individual benefits of early ART [26, 27] and differentiated models of care, including same-day [28] and community provision of ART [29], could alleviate the burden of seeking ART, thus allowing patients to initiate treatment earlier potentially while in the acute phase of infection [30–32].

### Overview of the AHRI Population-Based Cohort

Since 2004, AHRI has conducted annual population-based HIV testing among all consenting adults aged 15 years or older in a community immediately adjacent to the ANRS TasP trial area [23]. The AHRI cohort constitutes one of the world’s largest population-based longitudinal HIV cohorts and has measured the population trends in directly measured HIV incidence and quantified important socio-demographic, behavioural, and contextual determinants of newly acquired HIV infections [23]. Both the ANRS TasP trial and the AHRI population-based cohort share a very similar *modus operandi* based on the gold-standard approach of actively enrolling and following up a complete population and observing individual HIV seroconversions in those participants who were initially observed to be HIV-uninfected. The main difference between the two cohorts is that the AHRI population cohort conducts annual home-based testing, whereas the ANRS TasP trial conducted testing at 6-month intervals. The other major difference is that the period of follow-up is longer in the AHRI population cohort (> 14 years versus an average of 2.3 years in the ANRS TasP trial), encompassing the full period of ART scale-up.

A major strength of population-based cohorts that have enrolled and prospectively followed complete populations over decades is that representative knowledge (both with respect to disease outcomes but also on a dynamic suite of socio-demographic-, societal-, and community-level risk factors) is gained on all participants over time irrespective of whether individuals attend care. Such designs provide a strong basis for causal inference as well as a good standpoint from which to quantify the population-level impacts of interventions. The findings are therefore not subject to many of the biases commonly inherent in clinical studies based on patients who choose (and are able) to attend clinic or on a pre-selected sample of individuals who might differ from the population in ways that are difficult to evaluate. Moreover, because changes in measures like household wealth and sexual behaviour are systematically measured over time for each individual, it means that these measures can be used to explicitly rule out alternative explanations of the relationships observed, and that any findings are not subject to the pitfalls of ecological fallacy. The rich data measured in these population-based studies have high external validity which also provides opportunities for quasi-experimental study designs, such as regression discontinuity and instrumental variable designs, to control for all unobserved confounding [33•, 34]. In the same way that the ongoing population-based cohorts like the Framingham Heart Study have been able to generate important insights into the underlying risk factors for cardio-vascular disease [35], so too have ongoing population cohorts such as the AHRI cohort in South Africa [23] and the Rakai cohort in Uganda [36, 37] generated profound epidemiological insights into the risk factors, trajectory of epidemics, mechanisms and underlying causal risk factors, and pathways to HIV acquisition.

By the end of 2017, the AHRI population-based cohort contained ~ 105, 000 person-years of observation and ~ 3500 directly observed HIV seroconversions [38]. These large sample sizes taken from a complete population followed longitudinally for well over a decade permit powerful statistical inference. This in turn can facilitate a deep and nuanced understanding of underlying causal risk factors and processes and a quantification of dynamic incidence patterns among different population sub-strata allowing for identification of particularly vulnerable sub-groups [39–42].

### Key Findings from the AHRI Population-Based Cohort

**Fig. 1:**
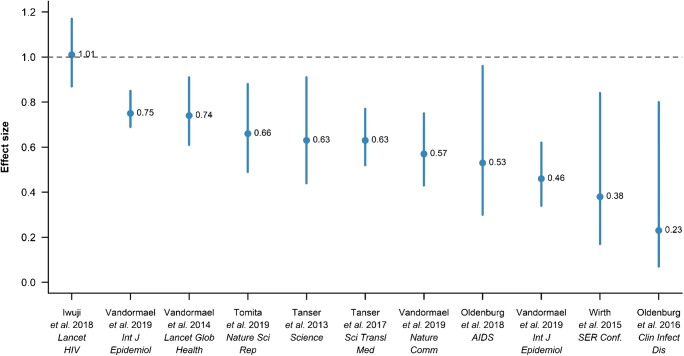
Summary of findings of the ANRS 12249 cluster-randomized trial (most left-hand point [3••]) as well as the results of ten controlled studies from the ongoing AHRI longitudinal population cohort [38, 43•, 44••, 45–50]. Both the ANRS TasP trial and the ongoing population-based cohort follow a similar *modus operandi* and utilize the gold-standard approach of enrolling and actively following up a complete population over time and directly measuring individual HIV seroconversions in those individuals who are initially observed to be HIV-uninfected. The studies in the population cohort have quantified the real-world, long-term impacts of expanding ART provision on reduction HIV incidence (and incidence-derived metrics) [38, 43•, 44••, 45–49]. One study also quantified the risk of expanding ART provision on newly diagnosed TB infection [50]. Further details of these studies are provided in Table 1. The studies utilize one of the world’s largest ongoing population-based cohorts that has measured the socio-demographic, behavioural, and contextual determinants of HIV incidence as well as the population trends over >14 years. The duration of follow-up of the population cohort encompasses the period both immediately before and after the scale-up of ART allowing for strong experimental separation in ART exposure (i.e., large differences in ART coverage) across time and space, within (and across) couples and households, as well as between different population sub-groups. The studies include quasi-experimental designs, such as regression discontinuity and instrumental variable designs

Figure 1 (accompanied by a more detailed description in Table 1) summarizes the ANRS TasP trial results [3••] as well as ten recent, controlled studies from the ongoing population-based cohort [38, 43•, 44••, 45–50]. These studies have meticulously quantified the real-world, long-term impacts of expanding ART provision on reduction in risk of new HIV infection across communities [44••, 45], within households [46, 47], within couples [49], and across the general population [38, 43•, 48]. One recent study also quantified the risk of expansion of ART provision on newly diagnosed TB infection [50]. In the population-based cohort, the duration of follow-up encompasses the period immediately both before and after the scale-up of ART, allowing for strong experimental separation among different population sub-groups in respect of ART exposure (i.e., large differences in ART coverage). These studies have exploited this heterogeneity in ART exposure and viral suppression across individuals and communities, within couples and households, and over time and space, for robust causal inference. Using this variation, the studies demonstrate consistently strong evidence for the preventative benefits of ART, using diverse methods and statistical models and explicitly controlling for well-known predictors of HIV incidence (Fig. 1, Table 1).

**Table 1:** Summary of findings of the ANRS 12249 cluster-randomized trial as well as the results of 10 recently published controlled studies from the ongoing longitudinal population cohort (in order of ascending date).

Study	Period and participants	Objectives	Results	Sample size (*N*); effect estimate (95% CI); *P* value	Differential in ART coverage or other indicators	Conclusion
Tanser et al. [44••]	2004–2011; women aged 15–49 years, men aged 15–54 years	To measure the effect of community-level ART coverage on HIV incidence, controlling for multiple socio-demographic, behavioural, and community variables.	An HIV-uninfected individual living in a community with high ART coverage (30 to 40% of all HIV-infected individuals on ART) was 37% less likely to acquire HIV than someone living in a community where ART coverage was low (< 10% of all HIV-infected individuals on ART).	*N* = 16,667; aHR = 0.63 (0.44–0.91); *P* = 0.013	30–40% vs. < 10%	Population-level reductions in the transmission of HIV can be achieved in nurse-led, devolved, public-sector ART programs in rural sub-Saharan African settings where complete coverage of therapy under existing treatment guidelines has not yet been attained.
Vandormael et al. [46]	2004–2012; women aged 15–49 years, men aged 15–54 years	To measure the effect of ART usage in the household on HIV incidence, controlling for multiple socio-demographic, behavioural, and community variables.	An HIV-uninfected individual living in a household with high opposite-sex ART coverage (50 to 60%) was 26% less likely to acquire HIV than someone living in a household with a low opposite-sex ART coverage (< 10%).	*N* = 14,505; aHR = 0.74 (0.61–0.91); *P* < 0.05	50–60% vs. < 10%	Results provide further evidence that ART significantly reduces the risk of onward transmission of HIV in a real-world setting. Awareness that ART can prevent transmission to co-resident sexual partners could be a powerful motivator for HIV testing and ART uptake, retention, and adherence.
Wirth et al. [45]	2004–2011; women aged 15–49 years, men aged 15–54 years	To evaluate the impact of community ART coverage on HIV incidence risk using an instrumental variable (IV) approach. The IV approach was used to account for the possibility that individuals living in high ART coverage areas may systematically differ from those in low ART coverage areas even after controlling for multiple predictors of infection.	In the IV-adjusted model, persons in communities with > 40% ART coverage were 62% less likely to acquire HIV infection than persons in communities with < 10% ART coverage.	Person-years = 53,605; aHR = 0.38 (0.17–0.84); *P* < 0.001	> 40% vs. < 10%	The findings indicate that the effect of community-level ART coverage on HIV incidence was not only robust to unmeasured confounding but may be stronger than previously reported in Tanser et al. [44••]
Oldenburg et al. [49]	2005–2013; women and men aged > 15 years	To evaluate the preventative impact of ART on HIV incidence in stable sero-discordant couples, controlling for multiple socio-demographic, behavioural, and community variables.	Use of ART was associated with a 77% decrease in HIV incidence risk among sero-discordant couples.	*N* = 17,016 and *n* = 2042 (discordant couples); aHR = 0.23 (0.07–0.80); *P* = 0.02	On ART vs. not on ART	ART initiation was associated with a large reduction in HIV incidence in sero-discordant couples in rural KwaZulu-Natal.
Tanser et al. [48]	2011–2015; women aged 15–49 years, men aged 15–54 years	To empirically quantify the relationship between a range of population viral load (PVL) measures and the prospective risk of HIV incidence among participants who were HIV-negative at baseline. Analyses were controlled for multiple socio-demographic, behavioural, and community variables.	Prospective HIV incidence in communities where the population prevalence of detectable viremia in the 1st quartile (< 12%) was 37% lower compared to communities in the 4th quartile (> 19%).	*N* = 8732; aHR = 0.63 (0.52–0.77); *P* < 0.001	Population prevalence of detectable viremia > 19% (4th quantile) vs. 12% (1st quantile)	Results show a clear relationship between PVL measures such as the population prevalence of detectable viremia and prospective incidence of HIV infection. PVL indices could play a key role in targeting and monitoring interventions in the most vulnerable communities where the future rate of new HIV infections is likely to be highest.
Iwuji et al. [3••]	2012–2016; resident men and women aged > 16 years	To investigate the effect of universal test and treat on HIV incidence using a cluster-randomized trial. The intervention group received immediate ART initiation upon HIV diagnosis. The control group had ART initiation at CD4 T cell counts < 350 and < 500 cells/μl upon HIV diagnosis (following national eligibility guidelines).	HIV incidence was 2.11 per 100 person-years (95% CI 1.84–2.39) in the intervention group and 2.27 per 100 person-years (2.00–2.54) in the control group.	*N* = 26,518; aHR = 1.01 (0.87–1.17); *P* = 0.89	45% ART coverage (in the treatment group) vs. 43% (in the control group)	There was no difference in HIV incidence between the intervention and control groups. Absence of a lowering of HIV incidence in universal test and treat clusters occurred as a consequence of there being no difference in ART coverage and population-level viral suppression between control and intervention communities.
Oldenburg et al. [47]	2007–2011, women and men aged > 15 years	To investigate the effect of immediate vs. delayed ART on HIV incidence among household members. The study used a quasi-experimental approach (regression discontinuity), which is designed to improve causal inference in nonrandomized studies.	Compared with delayed ART initiation, immediate initiation reduced HIV incidence in households by 47% and by 32–60% in alternate specifications of the model.	*N* = 4115; aHR = 0.53 (0.30–0.96); *P* < 0.05	Threshold of 200 CD4+ count cell/μl used to determine immediate vs. delayed initiation	This study demonstrates for the first time causally some of the spill-over effects that contribute to the population impact of HIV treatment on HIV incidence.
Vandormael et al. [38]	2005–2017; women aged 15–49 years, men aged 15–54 years	To quantify trends in the incidence to mortality ratio (IMR) between 2005 and 2017. The UNAIDS has proposed IMR as a key measure of epidemic control [51]. Epidemic control is achieved when the ratio of new infections to the number of all-cause HIV-related deaths falls below 1.	The observed IMR peaked at 5.74 in 2013 before declining to 4.06 in 2017. Bootstrapped estimates show an IMR reduction of 25% during this period.	*N* = 22,758 (HIV^−^ cohort) and *N* = 13,460 (HIV^+^ cohort); IMR = 0.75 (0.69–0.85); *P* < 0.05	ART coverage increased from 2% in 2005 (CD4+ count < 200 cells/μl) to 30% in 2011 (< 350 cells/μl) to 47% in 2016 (all eligible) and to 46% in 2017.	The results show impressive progress toward HIV epidemic control in the study area. However, the IMR epidemic threshold < 1 was not reached in 2017. Progress is off track for 2020 targets set by the UNAIDS.
Vandormael et al. [38]	2005–2017; women aged 15–49 years, men aged 15–54 years	To quantify trends in the incidence to prevalence ratio (IPR) between 2005 and 2017. The IPR is another metric of HIV epidemic control proposed by UNAIDS. Epidemic control is achieved when there is less than one new HIV infection over a 33-year period on ART. The average survival time of a newly infected person on ART is 33 years, which translates into 1/33 or 3 new infections per 100 people living with HIV per year.^[51]^	The IPR declined from 0.144 in 2012 to 0.075 in 2017. Bootstrapped estimates show an IPR reduction of 54% during this period.	*N* = 39,735 (HIV prevalence); *N* = 22,758 (HIV^−^ cohort) IPR = 0.46 (0.34–0.62); *P* < 0.05	ART coverage increased from 2% in 2005 (CD4+ count < 200 cells/μl) to 30% in 2011 (< 350 cells/μl) to 47% in 2016 (all eligible) and to 46% in 2017.	The decline in this metric indicates further progress toward HIV epidemic control in the study area. However, the epidemic threshold of < 0.03 was not reached in 2017. Progress is off track for 2020 targets set by the UNAIDS.
Tomita et al. [50]	2009–2015; men and women aged > 15 years	To quantify the impact of community coverage of ART on recently diagnosed TB disease, controlling for multiple socio-demographic, behavioural, and community variables.	Living in a community with ART coverage ≥ 50% was associated with a 34% decrease in the odds of recently diagnosed TB vs. living in a community with ART coverage < 50%.	*N* = 41,812; aOR = 0.66 (0.49–0.88); *P* = 0.005	ART coverage ≥50% vs. < 50%	Results indicate the potential benefit of increased community ART coverage in lowering the risk of active tuberculosis highlighting the need to prioritize the expansion of such effective population interventions targeting high-risk areas.
Vandormael et al. [43•]	2005–2017; women aged 15–49 years, men aged 15–54 years	To quantify trends in the population-wide HIV incidence following ART scale-up, controlling for multiple socio-demographic, behavioural, and community variables.	The HIV incidence rate declined from 3.94 (95% CI 3.37–4.60) to 2.25 (1.79–2.83) events per 100 person-years between 2012 and 2017—a reduction of 43%.	*N* = 22,239; IRR = 0.57 (0.43–0.75); *P* < 0.001	ART coverage increased from 2% in 2005 (CD4+ count < 200 cells/μl) to 30% in 2011 (< 350 cells/μl) to 47% in 2016 (all eligible) and to 46% in 2017.	The study shows robust evidence of large HIV incidence declines among men and women, which are consistent with the scale-up of ART and VMMC services.

*ART*, antiretroviral therapy; *aHR*, adjusted hazard ratio; *aOR*, adjusted odds ratio; *IV*, instrumental variable; *IMR*, incidence to mortality ratio; *IPR*, incidence to prevalence ratio; *IRR*, incidence rate ratio; *VMMC*, voluntary medical male circumcision

These studies have quantified the real-world, long-term impacts of expanding ART provision on reduction in risk of new HIV infection across different communities, within households, within sero-discordant couples, and in the general population

For example, the first study from the population cohort quantifying the treatment as prevention effect found that a 1% increase in ART coverage in the surrounding community is independently associated with an average 1.4% decline in an individual’s risk of acquisition of HIV infection (adjusted hazard ratio (aHR) = 0.986) [44••]. The results of the study and implications for treatment as prevention at the time are discussed in two commentaries [52, 53]. Other findings from this population cohort demonstrate, for example, that within sero-discordant couples, use of ART is associated with a 77% decrease in HIV incidence [49]. Within households, an HIV-uninfected individual in a household characterized by high opposite-sex ART coverage is 26% less likely to acquire HIV than someone living in a household with a low opposite-sex ART coverage [46] and compared with delayed ART initiation, immediate initiation of ART reduced HIV incidence in households by 47% [47]. At a community-level, every 1% increase in the proportion of an entire community having a detectable virus is independently associated with a 6.3% prospective increase in risk of HIV acquisition for HIV-negative individuals living in that community [48].

**Fig. 2:**
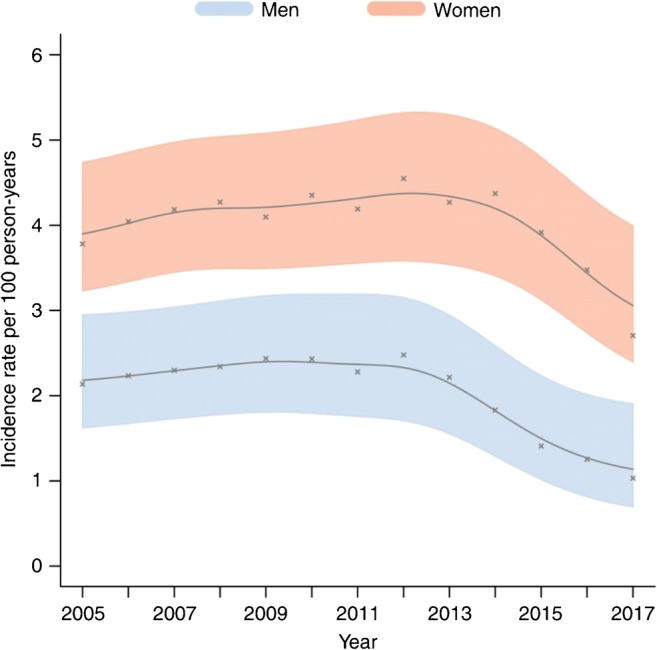
Population trends in HIV incidence with 95% confidence intervals (CIs) between 2005 and 2017 in the AHRI population cohort. Male and female HIV incidence declined substantially after 2012 and 2014, respectively, with an overall population decline of 43% between 2012 and 2017. Reproduced from Vandormael et al. (2019) [43•] under a creative commons licence (http://creativecommons.org/licenses/by/4.0/)

At a population level, overall HIV incidence between 2012 and 2017 declined dramatically by 43% (Fig. 2) [43•]. Consistent with treatment as prevention playing a major role in this population-level reduction, HIV incidence declined among both circumcised and uncircumcised men. Moreover, men experienced earlier and larger HIV incidence declines than women, consistent with higher ART coverage in women. Specifically, male incidence declined by 59%, from 2.5 to 1.0 sero-conversion events per 100 person-years, which coincided with female ART coverage surpassing 35% in 2012 and VMMC scale-up in 2009. There was a 37% reduction in female HIV incidence between 2014 and 2017, from 4.9 to 3.1 sero-conversion events per 100 person-years, which occurred after male ART coverage reached 35% [43•]. While overall progress is off track to meet the 2020 reduction targets set by the UNAIDS [51], a recent paper documented impressive progress toward HIV epidemic control in this population [38]. Among men, the incidence to mortality ratio peaked at 4.1 in 2013 before dropping to 3.1 in 2017 (a 24% reduction) while the female incidence to mortality ratio climbed to as high as 6.4 in 2013 before dropping to 4.3 in 2017 (a 33% reduction). Between 2012 and 2017, the male-incidence to female-prevalence ratio declined from 0.05 to 0.02. Compared with men, however, the female-incidence to male-prevalence ratio was markedly higher and fell from 0.24 to 0.13 during the same period [38]. This result, when coupled with the higher HIV incidence, incidence to mortality ratio, and HIV prevalence among women, confirms the disproportionate burden of HIV being experienced by women relative to men in sub-Saharan Africa. Treatment for HIV is also associated with secondary preventative benefits for TB infection and shows a 34% reduction in the risk of newly diagnosed TB infection to an individual living in a community with ≥ 50% ART coverage (adjusted odds ratio (aOR) = 0.66, 95% CI 0.49–0.88) [50].

The results of these studies (Table 1, Fig. 1) are epidemiologically plausible and clear, measurable reductions in HIV incidence and incidence-derived metrics were consistently found across all studies. The findings were robust to different model specifications, different age-eligibility criteria, differing methods of constructing “communities,” and the inclusion of differing control variables (including being robust to changes in sexual behaviour, for example). Further, methods to impute the date of HIV seroconversion were systematically investigated and the results were found to be robust to participant self-selection associated with missed test dates and drop-out [54, 55]. It is thus unlikely that any collection of systematic biases could consistently and simultaneously explain the findings across the different studies conducted within households, couples, communities, population sub-groups, genders, and using differing outcome metrics (and in one case, the outcome of a different disease—i.e., newly diagnosed TB infection). Nevertheless, the possibility of the existence of such a pervasive unidirectional residual confounding effect—however unlikely—should be acknowledged. To rule out the possibility of residual confounding, two quasi-experimental study designs [45, 47] were implemented using instrumental variable (IV) and regression discontinuity (RD) designs (Table 1). They do this by quasi-randomly assigning individuals to intervention vs. control groups, leveraging randomness induced by policy, practice, or natural events [56–61]. The quasi-experimental studies [45, 47] confirmed a large real-world treatment as prevention effect that could not have been explained by the influence of any observed or unobserved factors. The Wirth et al. [45] analysis not only confirmed the previous findings (and therefore demonstrated that the result was robust to the effect of unmeasured confounding) but also suggested that the effect of community-level ART coverage on HIV incidence may be even greater than previously estimated in the paper published in *Science* [44••].

## Conclusion

All of the evidence from large, population-based studies (inclusive of the ANRS TasP trial) in this setting is remarkably consistent—i.e., higher ART coverage and population viral suppression were repeatedly associated with large, measurable decreases in HIV incidence. Despite increases in population levels of viral suppression in both arms, the offer of UTT in ANRS TasP trial did not induce differences in viral suppression between intervention and control communities and thus the trial could not demonstrate a relative reduction in HIV incidence in the intervention communities. As one of the world’s largest ongoing HIV incidence cohorts and spanning the period both immediately before and after the scale-up of antiretroviral therapy, the AHRI population cohort allowed for strong experimental separation in ART exposure (i.e., large differences in ART coverage) and viral suppression between different population sub-groups. The studies summarized in this commentary exploited this heterogeneity in ART exposure across individuals and communities, within couples and households, and over time and space, for a robust quantification of the treatment as prevention effect in a real-life setting.

In summary, the recent evidence from controlled, population-based studies in this typical rural African population demonstrates that expanded provision of ART has substantially and consistently reduced the risk of onward transmission at multiple levels, which has plausibly contributed in a major way toward the dramatic 43% decline in population-level HIV incidence. Going forward, however, the incremental gains in incidence reduction are likely going to be harder to achieve. The outcome of the ANRS TasP trial constitutes a powerful null finding with important lessons for overcoming implementation challenges in the population delivery of ART. This finding does not imply lack of ART effectiveness in preventing the onward transmission of HIV nor the inability to reduce population-level HIV incidence. Rather, it demonstrates that large increases in ART coverage over current levels will require health systems innovations to attract people living with HIV in early stages of the disease to participate in HIV treatment. Such innovations and new approaches are required for the true potential of UTT to be realized. Attaining epidemic control will require overcoming existing implementation barriers to the continued expansion of ART accompanied by the provision of other primary prevention measures.
